# A Study to Inform the Design of a National Multicentre Randomised Controlled Trial to Evaluate If Reducing Serum Phosphate to Normal Levels Improves Clinical Outcomes including Mortality, Cardiovascular Events, Bone Pain, or Fracture in Patients on Dialysis

**DOI:** 10.1155/2015/579434

**Published:** 2015-08-23

**Authors:** Ramya Bhargava, Philip A. Kalra, Paul Brenchley, Helen Hurst, Alastair Hutchison

**Affiliations:** ^1^Institute of Cardio-Vascular Sciences, Faculty of Medical and Human Sciences, University of Manchester, Manchester M13 9PL, UK; ^2^Manchester Royal Infirmary, Central Manchester University Hospitals NHS Foundation Trust, Manchester Academic Health Science Centre, Manchester M13 9WL, UK; ^3^Salford Royal Hospitals NHS Foundation Trust, Salford M6 8HD, UK; ^4^Institute of Population Studies, Faculty of Medical and Human Sciences, University of Manchester, Manchester M13 9PL, UK

## Abstract

*Background*. Retrospective, observational studies link high phosphate with mortality in dialysis patients. This generates research hypotheses but does not establish “cause-and-effect.” A large randomised controlled trial (RCT) of about 3000 patients randomised 50 : 50 to lower or higher phosphate ranges is required to answer the key question: does reducing phosphate levels improve clinical outcomes? Whether such a trial is technically possible is unknown; therefore, a study is necessary to inform the design and conduct of a future, definitive trial. *Methodology*. Dual centre prospective parallel group study: 100 dialysis patients randomized to lower (phosphate target 0.8 to 1.4 mmol/L) or higher range group (1.8 to 2.4 mmol/L). Non-calcium-containing phosphate binders and questionnaires will be used to achieve target phosphate. Primary endpoint: percentage successfully titrated to required range and percentage maintained in these groups over the maintenance period. Secondary endpoints: consent rate, drop-out rates, and cardiovascular events. *Discussion*. This study will inform design of a large definitive trial of the effect of phosphate on mortality and cardiovascular events in dialysis patients. If phosphate lowering improves outcomes, we would be reassured of the validity of this clinical practice. If, on the other hand, there is no improvement, a reassessment of resource allocation to therapies proven to improve outcomes will result. *Trial Registration Number*. This trial is registered with ISRCTN registration number ISRCTN24741445.

## 1. Background

Dialysis requires more “self-management” than any other medical treatment to control risk factors associated with increased mortality. A patient starting dialysis, aged 60 years, may be informed that they have a 50% chance of surviving five years [1], but observational studies suggest this can be improved by controlling intake of fluids, salt, fat, phosphate, and potassium, attending regularly for dialysis, monitoring blood pressure, cholesterol, phosphate, and haemoglobin, and taking prescribed medication to control these factors, plus weekly injections to improve haemoglobin [2]. Few people are able to understand and manage all aspects simultaneously, and their relative importance is unknown. Meaningful discussion between clinician and patient about these issues, and phosphate control in particular, is currently not possible and unsurprisingly adherence to prescribed regimens is poor [3, 4]. We will address the significance of one risk factor for mortality in dialysis patients—serum phosphate—to facilitate and inform future patient-clinician discussions and enhance the shared decision making process.

The normal serum phosphate is 0.8 to 1.4 mmol/L. Serum phosphate increases in chronic kidney disease and by the time the patients are on dialysis, high serum phosphate is found in more than 40% of dialysis patients and is linked with a 40–100% increased mortality risk in retrospective, observational studies [5, 6]. Opinion-based serum phosphate of less than 1.7 mmol/L is the target for treatment in dialysis patients [7]. 27 observational studies were included in a meta-analysis which examined the relationship between dysregulated mineral metabolism and all-cause or cardiovascular mortality or cardiovascular events in patients with chronic kidney disease (CKD) or end-stage renal disease (ESRD, which is when they need to start dialysis treatment) [8]. Though there were limitations in the analysis noted by the authors due to the low number of studies included and the quality of the data obtained from them, a greater risk of all-cause and cardiovascular mortality was seen with elevated phosphate concentrations. The Dialysis Outcomes and Practice Patterns Study (DOPPS), a prospective cohort study in 25,588 patients with ESRD receiving haemodialysis, showed an increased risk of cardiovascular mortality with serum phosphate concentrations of 5.1–5.5 mg/dL (1.6–1.78 mmol/L) and an increase in all-cause mortality at serum concentrations over 6.0 mg/dL (1.94 mmol/L) [9]. It is recognised that such studies are useful for generating research hypotheses but cannot definitively establish “cause/effect” relationships. Consequently it is believed important to control phosphate, but whether this improves patient outcomes remains unknown, since no randomised interventional trials have been undertaken.

Dietary control and dialysis are insufficient to normalise phosphate, and tablets are required to bind phosphate in the gut. Binders make phosphate insoluble, preventing absorption from the intestinal lumen. The available phosphate binders can be broadly classified into calcium containing phosphate binders like calcium carbonate and calcium acetate and non-calcium containing binders like lanthanum (Fosrenol—trade name) and sevelamer (Renvela and Renagel—trade names for sevelamer carbonate and sevelamer hydrochloride, resp.) and aluminium containing and iron containing phosphate binders. This classification is important because several small interventional randomised controlled trials have looked at difference in clinical outcomes between calcium containing phosphate binders and the common non-calcium containing ones like lanthanum and sevelamer. Jamal et al. published a meta-analysis of 11 such studies, 9 of them in dialysis patients, and compared outcomes between patients with chronic kidney disease taking calcium-based phosphate binders and those taking non-calcium-based binders. They concluded that non-calcium-based phosphate binders were associated with a decreased risk of all-cause mortality compared with calcium-based phosphate binders in patients with chronic kidney disease [10]. Multiple clinical trials have further shown an increase in coronary artery calcification with calcium-based binders compared to non-calcium-based binders [11–18]. There is an increasing acceptance among clinicians that calcium containing phosphate binder is not optimum therapy and has resulted in greater use of non-calcium containing binders which are ten times more expensive than the calcium containing binders.

A study of patients' perspectives of phosphate binding medication identified gaps in understanding of the concept of phosphate control and the role of medication [3, 19]. Even when binders are taken correctly, achieving normal phosphate levels is difficult, and more than 25% of patients remain above the current opinion-based target range of 1.1–1.7 mmol/L, and more than 40% are above the true normal limit of 1.4 mmol/L [20, 21]. Despite the publication of multiple guidelines, there is a significant gap between the recommendations and the serum phosphate concentrations achieved by patients in clinical practice [22]. The important contributing causes to the lack of effective phosphate control are nonadherence to a low phosphate diet and to phosphate binder medication [23]. Despite the investment in expensive binder medication (up to £3,000 per patient/per annum, 30% of the 27000 prevalent dialysis patients in UK [24], works out to more than £24 m per annum) and large pill burden (up to 15 pills with meals daily in some cases) with significant gastrointestinal side-effects [25], we do not know if lowering serum phosphate is of benefit [26]. A review of all available evidence by the international “Kidney Disease Improving Global Outcomes (KDIGO)” expert group [7] concluded, “*the extensive review… exposed significant gaps in our knowledge… robust studies of a large sample size addressing the following issues should be given priority: Does lowering phosphate… improve clinical outcomes including mortality?*” Our trial will examine the feasibility of conducting such a study of large sample size, which we hope will ultimately answer the questions posed by KDIGO.

## 2. Methods/Design

### 2.1. Primary Endpoint

The primary endpoint is the percentage of study participants achieving, and being maintained within, the higher and lower target ranges for phosphate, over the duration of the maintenance phase of the study.

### 2.2. Secondary Endpoints

The secondary endpoints are the following:

1. Percentage of Greater Manchester kidney physicians agreeing to enter patients into a study which includes a “higher range” group.
2. Percentage of eligible invited participants willing to be randomised into a study which includes a “higher range” group.
3. Percentage of participants achieving consistent control of serum phosphate in each group over a 10-month maintenance period.
4. Drop-out rate from the study due to adverse events, kidney transplantation, intercurrent illness, and death. These numbers will inform the power calculation for the larger national study.
5. Pill burden per participant required to control serum phosphate.
6. Adherence to therapy.
7. Number of participants willing to participate in “Communicare” patient support programme.
8. Mean symptom score assessed by Pittsburgh Dialysis Symptoms Index.
9. Incidence of major vascular events, defined as nonfatal myocardial infarction or any cardiac death, any stroke, or any arterial revascularisation excluding dialysis access procedures (expected mortality of around 14% per annum in patients on dialysis).

### 2.3. Design

The design is a dual-centre, pan-Manchester prospective randomised parallel group study, with titration to target (2 months) and maintenance phase (10 months).

### 2.4. Total Number of Participants Planned

The total number of participants planned is 100 at randomisation (up to 300 at consent).

### 2.5. Setting

The study will be conducted across two large renal units in Greater Manchester which cover a population of about 3.2 million. Prevalent dialysis patients will be recruited from the renal units and their associated satellite dialysis units which give a target dialysis population of about 1100 patients with 900 patients on haemodialysis.

### 2.6. Ethics, Informed Consent, and Safety

Documented approval has been obtained from appropriate ethics committee and the CMFT Research and Development Department. The study conforms to International Conference on Harmonization of good clinical practice guidelines and with the Declaration of Helsinki. Written informed consent will be obtained from each patient before any study-specific procedure takes place. Participation in the study and date of informed consent of patients will be documented appropriately in each patient's files. Safety Monitoring Committee is in place to review SAEs as is Trial Steering Committee to review progress and Trial Management Committee to oversee the conduct of the trial.

#### 2.6.1. Identifying Participants

The medical records of all the dialysis patients in the Greater Manchester area will be accessed by the study personnel that are also a part of the direct care team (some study personnel will be members of the direct care team). A retrospective screening of their previous serum phosphate levels (which would have been done as part of their routine monthly blood tests) will be completed. Patients with a mean level of >1.4 mmol/L over the past 3 months, and taking an oral phosphate binder, will be identified and flagged up.

#### 2.6.2. Inclusion Criteria

Inclusion criteria are as follows:

1. Male and female patients aged 30 years or above, on dialysis for at least 6 months (to ensure no recovery of renal function), and under the supervision across pan-Manchester sites. Patients less than 30 years of age have a low rate of vascular events and will not be recruited.
2. Serum phosphate level of 1.7 mmol/L or greater after wash-out (discontinuation) of previous phosphate binding medication.
3. Able to achieve Renal Association standards for quality of dialysis on the most recent test of dialysis efficacy. This would be a urea reduction ratio of 65%.
4. Able to communicate in English because “Communicare” package (the package is explained in Treatments) is currently available only in English.
5. Able to consent.

#### 2.6.3. Exclusion Criteria

Exclusion criteria are as follows:

1. Living donor renal transplant planned in the next 12 months.
2. Serum parathyroid hormone greater than 800 pg/mL (85 pmol/L) on 2 consecutive 2- or 3-monthly blood tests. Such patients probably have uncontrolled hyperparathyroidism which adversely influences serum phosphate levels and needs treatment in its own right.
3. Known intolerance of both oral sevelamer and lanthanum carbonate.
4. Medical history that might limit the individual's ability to take the trial treatments for the duration of the study (e.g., history of cancer other than nonmelanoma skin cancer or recent history of alcohol or substance misuse).

**Figure 1:**
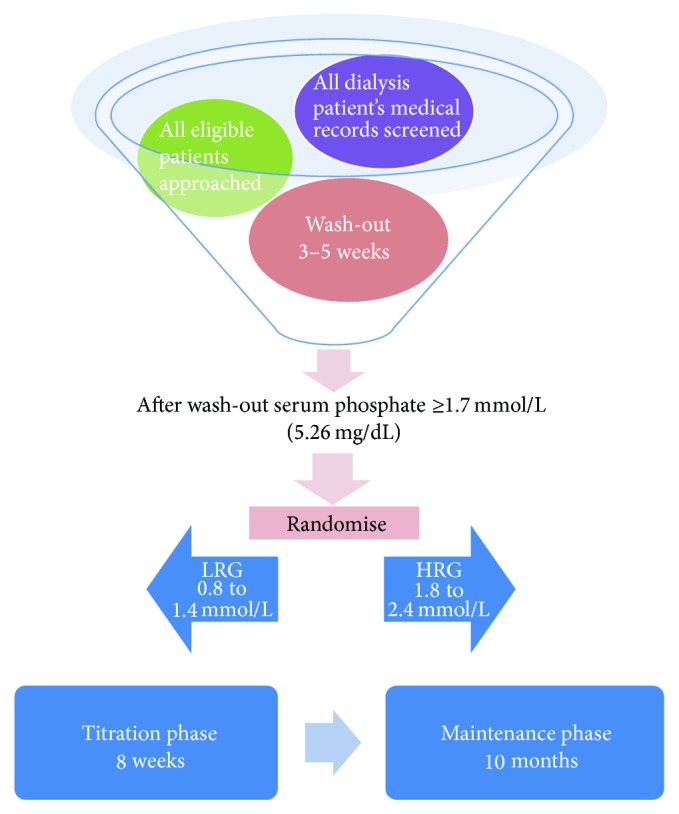
Recruitment and randomisation.

Figure 1 shows the outline of recruitment and randomisation.

### 2.7. Estimated Timeline

The trial is estimated to run over a period of 24 months. The duration of the study for each participant will be 13 months. Figure 1 illustrates the timeline for each participant in the study.

### 2.8. Treatments

Prior to randomisation, the number of potentially eligible participants will be identified (medical records) and each will be sent the Participant Information Sheet with an invitation to attend a screening clinic appointment. Written informed consent will be sought from those who attend the appointment and are willing to participate (−3 weeks). The ratio of “eligible” to “willing” will be recorded. Consenting participants will complete the “Pittsburgh Dialysis Symptom Index” [27] to identify baseline symptom score. They will undergo an assessment of adherence at baseline using modified Basel Assessment of Adherence Scale for ImmunoSuppressives (BAASIS). Patients whose average serum phosphate is more than or equal to 1.7 mmol/L despite ongoing therapy with phosphate binders will be randomised. Patients whose average serum phosphate is less than 1.7 mmol/L, and who are taking phosphate binders, will enter a 3- to 5-week “wash-out” period from their previous phosphate binder and receive standard dietary advice from a renal dietician. Serum lipids will be measured and treated according to UK Renal Association guidelines with a statin and/or ezetimibe [28]. Equivalent lipid levels would be required in the larger definitive trial to exclude the possibility of this influencing mortality. This is because two non-calcium containing phosphate binders, sevelamer and colestilan (Bindren—trade name), also reduce serum cholesterol [29]. Sevelamer has been observed to reduce absorption of advanced glycated end-products, bacterial toxins, and bile acids, suggesting that it may reduce inflammatory, oxidative, and atherogenic stimuli in addition to its lowering of serum phosphate [30]. Provided that serum phosphate level rises to greater than or equal to 1.7 mmol/L after wash-out, each participant will be randomised to either the lower range (LRG) or higher range (HRG) phosphate group. Participants with phosphate of less than 1.7 mmol/L after wash-out will not continue the study. This minimises the possibility of an individual being randomised to HRG but whose phosphate level will not reach 1.7 mmol/L.

Those randomised to LRG will undergo a stepped approach to aid achievement of the lower range phosphate target. The treatment for each study visit is summarised in Table 1.

#### 2.8.1. Communicare Package

During each of their dialysis sessions in the first week after randomisation, participants will be given access to, and encouraged to utilise, the “Communicare” online patient adherence support programme. This comprises a patient questionnaire, developed by the London School of Behavioural Science, which highlights individual patient information needs and concerns related to taking phosphate binding medication. Questionnaire results give participants access to online tailored, personalised (but reproducible and standardised) information or “Info Bytes” to help address the concerns they have about taking phosphate binding medication. The package also provides training for the study staff to enable them to discuss phosphate control knowledgeably with participants. There is a paper version of the “Communicare” package which can also be used.

The LRG participants will be able to access the Communicare online support programme at any time but will be specifically encouraged to do so again during dialysis session in month 4 and month 8.

#### 2.8.2. Oral Phosphate Binders

They will recommence oral phosphate binding medication with either lanthanum carbonate or sevelamer (either carbonate or hydrochloride), titrated on a weekly basis with meals to achieve serum phosphate of 0.8 to 1.4 mmol/L in 8 weeks' time.

Since sevelamer reduces serum lipid levels, those individuals taking a statin for cholesterol reduction may require dose adjustment. The dose adjustment of statin will be completed by the study clinicians according to their clinical judgement, with a view to maintaining the serum cholesterol according to the standards set by the Renal Association.

#### 2.8.3. Assessment of Adherence

Adherence will be assessed by the modified BAASIS questionnaire [31, 32]. This is a validated questionnaire which we have modified to reflect phosphate binders instead of immunosuppressants. It comprises 4 questions which the patient answers once every 4 weeks. This questionnaire has been validated in kidney transplant patients [33] on immunosuppressive medications and in HIV patients who are on antiretroviral medications. Both of these groups need to take their medications on a regular timely basis. This criterion applies to the administration of phosphate binders which need to be taken regularly and with each meal to be effective.

All patients will have their adherence assessed at baseline; only the patients randomised to the LRG will continue to have their adherence assessed once every 4 weeks.

Those randomised to HRG will recommence oral phosphate binding medication in the weeks following randomisation, with either lanthanum carbonate or sevelamer, titrated on a weekly basis to achieve a serum phosphate level of 1.8–2.4 mmol/L. We anticipate that some will require no phosphate binding medication to achieve this. The treatment for each study visit is summarised in Table 2.

The participants will have a range of licensed phosphate binding medication to choose from—chewable tablets (lanthanum—Fosrenol), tablets to be swallowed (sevelamer—Renvela, Renagel), and granules that can be mixed with water and consumed (Fosrenol, Renvela)—as first-line therapy. Changes to the phosphate binders and the cholesterol medications during the study will be documented in a drug dosing diary which will be carried by the patient.

Phosphate in all participants will be monitored on a monthly basis from week 8 onwards, with medication adjustments as necessary to maintain results within range. All haemodialysis patients have blood taken routinely for biochemical and haematological measurements on a monthly basis; attempts will be made to ensure that the study blood tests coincide with the routine monthly blood tests; this ensures a reduction in the number of additional blood tests required by this study during the maintenance phase.

A blood sample will be collected at the consent visit, at randomisation, and every 12 weeks thereafter to measure the serum level of parathyroid hormone (PTH). All dialysis patients have this blood test once every three months as part of routine clinical care. Efforts will be made to ensure that the study blood test coincides with their routine test to minimise the number of extra blood tests.

Participants will be asked to gift an extra 5 mL of blood with every blood sample collected for parathyroid hormone. This will be stored in the biobank at the Renal Research Laboratories, Manchester Royal Infirmary, for future biomarker analysis. All participants will complete the Pittsburgh Dialysis Symptoms Index [27] again at month 6 and month 12 (study end).

Oral vitamin D dosage will be altered if necessary to ensure good control of serum calcium PTH [7].

### 2.9. Drug Dosing

All participants will be given a choice of phosphate binders to use. They will be commenced on one of the two non-calcium containing phosphate binders determined by their preference (chewable, swallowed, and granules). The dosage of the medication will be increased once a week during the titration phase. The target will be to achieve the desired range of serum phosphate. The changes to drugs and dosages done as part of the study will be recorded in a drug dosing diary which the patient will carry.

### 2.10. Dosing Schedule in the LRG (0.8 to 1.4 mmol/L)

Phosphate binders are prescribed in daily doses divided according to the estimated phosphate content of the meals. For some patients, this is three equal doses, whilst for others it might be two different daily doses. Standard practice is for this advice to be given by the prescribing physician or by a renal dietician.

The dosing schedule shown in Table 3 is only a guide and the phosphate binders can be dosed according to clinician judgement.

### 2.11. Dosing Schedule in the HRG (1.7 to 2.4 mmol/L)

It is expected that many patients in the HRG will not require a phosphate binder. However, at any stage during the study, oral non-calcium containing phosphate binder will be introduced if the serum phosphate level exceeds the upper limit of 2.4 mmol/L. The intention would be to reduce and stabilise the phosphate level within the specified range of 1.7–2.4 mmol/L.

Table 4 outlines the titration regime, but if a patient's serum phosphate exceeds 2.4 mmol/L for the first time during, for example, week 4, then they should commence titration at that point. Therefore this table describes “titration steps” rather than study weeks.

The participants in both of the groups are allowed to switch their phosphate binding medication at any stage in the study to one of the other non-calcium containing formulations. They will then be changed to their trial phosphate binding medication of choice at a dose determined by the trial physicians. The target is to achieve the desired range of serum phosphate with a dose and combination that is convenient to the patient in order to encourage adherence. The dose of the study medications can be altered to maintain the serum phosphates in the desired range in the maintenance period on a monthly basis, according to the discretion of the study clinicians. Each change will be documented and recorded.

### 2.12. Sample Size

No formal sample size calculation has been conducted given the exploratory and evaluative nature of the study. We will randomise 100 patients in total to the “lower range” group and “higher range” group. Assuming an overall attrition rate of 25% at 12 months, 75 patients will “complete” the data-monitoring period. This will be sufficient to allow the monitoring of logistical aspects of this study (such as recruitment, randomisation, and attrition).

### 2.13. Statistical Analysis and Data Collection

The analysis of this study will be largely descriptive. Detailed statistical evaluation will not be undertaken, and therefore the sample size is chosen to be representative of the Manchester dialysis population (1100 in total). It will be large enough to address the outcome measures but small enough to facilitate timely recruitment and follow-up.

We will be able to

1. estimate a confidence interval for the proportion of patients achieving consistent control of serum phosphate in each group,
2. estimate the major cardiovascular event (including death) rate and a confidence interval for this parameter,
3. observe and record time-to-event data.

These calculations will help to inform the sample size calculation for a multicentre randomised controlled trial, for which mortality and cardiovascular event rate are expected to be the primary outcome measures but only if sufficient events are observed. We will also monitor and summarise recruitment and attrition rates and collect data on reasons for study withdrawal.

Table 5 gives a list of assessment measures used for the different endpoints in the study.

## 3. Discussion

This study will inform design of a large definitive trial of the effect of phosphate on mortality and cardiovascular events in dialysis patients, starting 2016/17. If reduction of phosphate improves life expectancy, then both clinicians and patients will be better informed and will be able to address this issue more certainly and appropriately, despite current drawbacks of phosphate binding medication. The time, inconvenience, and expense associated with phosphate control would be justified. If there is no benefit to reducing phosphate to a prespecified range, then patients may be relieved of the burden of excessive binding medication and side-effects. Savings from reduced prescriptions could be redirected to develop other methods believed to improve patients' quality and quantity of life, for example, increased provision of home dialysis therapies, with benefits to NHS capital/revenue expenditure. A study which could definitively show a cause-effect relationship between serum phosphate levels and clinical outcomes like mortality and cardiovascular events will be a game-changer in the management of dialysis patients.

## Figures and Tables

**Table 1:** LRG—treatment for each study visit.

Visit number	Trial timeline/study visits	Blood tests (renal/liver profiles/cholesterol)	Blood test (PTH) + extra sample	Dietician advice	Pittsburgh Dialysis Symptoms Index	Communicare package	BAASIS	Other events
0	−6 weeks to −3 weeks							Potential participants identified from patient records Contact made; PIS provided

1	−3 weeks			✓				Consent Washout period commences

2	Randomisation week 0	✓	✓			✓		Randomisation Commence oral phosphate binding trial medication— sevelamer or lanthanum

3	1 week after randomisation	✓						Titrate trial medication and statins Discuss symptoms

4	2 weeks after randomisation	✓						Titrate trial medication and statins Discuss symptoms

5	3 Weeks after randomisation	✓						Titrate trial medication and statins Discuss symptoms

6	4 weeks after randomisation	✓					✓	Titrate trial medication and statins Discuss symptoms

7	5 weeks after randomisation	✓						Titrate trial medication and statins Discuss symptoms

8	6 weeks after randomisation	✓						Titrate trial medication and statins Discuss symptoms

9	7 weeks after randomisation	✓						Titrate trial medication and statins Discuss symptoms

10	8 weeks after randomisation	✓	✓				✓	Review medical history Discuss symptoms Titrate trial medication and statins if necessary

11	12 weeks after randomisation	✓				✓	✓	Review medical history Discuss symptoms Titrate trial medication and statins if necessary

12	16 weeks after randomisation	✓					✓	Review medical history Discuss symptoms Titrate trial medication and statins if necessary

13	20 weeks after randomisation	✓	✓		✓		✓	Review medical history Discuss symptoms Titrate trial medication and statins if necessary

14	24 weeks after randomisation	✓					✓	Review medical history Discuss symptoms Titrate trial medication and statins if necessary

15	28 weeks after randomisation	✓				✓	✓	Review medical history Discuss symptoms Titrate trial medication and statins if necessary

16	32 weeks after randomisation	✓	✓				✓	Review medical history Discuss symptoms Titrate trial medication and statins if necessary

17	36 weeks after randomisation	✓					✓	Review medical history Discuss symptoms Titrate trial medication and statins if necessary

18	40 weeks after randomisation	✓					✓	Review medical history Discuss symptoms Titrate trial medication and statins if necessary

19	44 weeks after randomisation	✓	✓				✓	Review medical history Discuss symptoms Titrate trial medication and statins if necessary

20	48 weeks after randomisation	✓					✓	Review medical history Discuss symptoms Titrate trial medication and statins if necessary

21	52 weeks after randomisation	✓					✓	Review medical history Discuss symptoms Patient's involvement in the trial ends

**Table 2:** HRG—treatment for each study visit.

Visit number	Trial timeline	Blood tests (renal/liver/cholesterol)	Blood test (PTH) + extra sample	Dietician advice	Pittsburgh Dialysis Symptoms Index	Other events
0	−6 weeks to −3 weeks					Potential participants identified from patient records Contact made; PIS supplied

1	−3 weeks			✓		Consent Washout period commences

2	Randomisation week 0	✓	✓			Randomisation Commence oral phosphate binding trial medication—sevelamer or lanthanum

3	1 week after randomisation	✓				Titrate trial medication and statins Discuss symptoms

4	2 weeks after randomisation	✓				Titrate trial medication and statins Discuss symptoms

5	3 weeks after randomisation	✓				Titrate trial medication and statins Discuss symptoms

6	4 weeks after randomisation	✓				Titrate trial medication and statins Discuss symptoms

7	5 weeks after randomisation	✓				Titrate trial medication and statins Discuss symptoms

8	6 weeks after randomisation	✓				Titrate trial medication and statins Discuss symptoms

9	7 weeks after randomisation	✓				Titrate trial medication and statins Discuss symptoms

10	8 weeks after randomisation	✓	✓			Review medical history Discuss symptom Titrate trial medication and statins if necessary

11	12 weeks after randomisation	✓				Review medical history Discuss symptoms Titrate trial medication and statins if necessary

12	16 weeks after randomisation	✓				Review medical history Discuss symptoms Titrate trial medication and statins if necessary

13	20 weeks after randomisation	✓	✓		✓	Review medical history Discuss symptoms Titrate trial medication and statins if necessary

14	24 weeks after randomisation	✓				Review medical history Discuss symptoms Titrate trial medication and statins if necessary

15	28 weeks after randomisation	✓				Review medical history Discuss symptoms Titrate trial medication and statins if necessary

16	32 weeks after randomisation	✓	✓			Review medical history Discuss symptoms Titrate trial medication and statins if necessary

17	36 weeks after randomisation	✓				Review medical history Discuss symptoms Titrate trial medication and statins if necessary

18	40 weeks after randomisation	✓				Review medical history Discuss symptoms Titrate trial medication and statins if necessary

19	44 weeks after randomisation	✓	✓			Review medical history Discuss symptoms Titrate trial medication and statins if necessary

20	48 weeks after randomisation	✓				Review medical history Discuss symptoms Titrate trial medication and statins if necessary

21	52 weeks after randomisation	✓				Review medical history Discuss symptoms Patient's involvement in the trial ends

**Table 3:** Dosing schedule in the LRG.

Titration step	Renvela	Renagel	Fosrenol	Other phosphate binder (sevelamer if previously on lanthanum and vice versa)
1	2.4 g per day in divided doses	2.4 g per day in divided doses	1.5 g per day in divided doses	

2 (increase if needed)	4.8 g per day in divided doses	4.8 g per day in divided doses	2.0 g per day in divided doses	

3 (increase if needed)	7.2 g per day in divided doses	7.2 g per day in divided doses	2.5 g per day in divided doses	

4 (increase if needed)	9.6 g per day in divided doses	9.6 g per day in divided doses	3 g per day in divided doses	

6 (if needed)	Continue at 9.6 g per day	Continue at 9.6 g per day	Continue at 3 g per day	Commence on week 3 dose

7 (increase if needed)	Continue at 9.6 g per day	Continue at 9.6 g per day	Continue at 3 g per day	Commence on week 4 dose

**Table 4:** Dosing schedule in the HRG.

Titration step	Renvela	Renagel	Fosrenol
1 if serum phosphate >2.4 mmol/L	1.6 g per day in divided doses	1.6 g per day in divided doses	500 mg per day

2 if serum phosphate >2.4 mmol/L	2.4 g per day in divided doses	2.4 g per day in divided doses	1.0 g per day in divided doses

3 if serum phosphate >2.4 mmol/L	3.2 g per day in divided doses	3.2 g per day in divided doses	1.5 g per day in divided doses

4 if serum phosphate >2.4 mmol/L	4.0 g per day in divided doses	4.0 g per day in divided doses	2.0 g per day in divided doses

6 (increase if needed)	4.8 g per day in divided doses	4.8 g per day in divided doses	2.5 g per day in divided doses

7 (increase if needed)	5.6 g per day in divided doses	5.6 g per day in divided doses	3.0 g per day in divided doses

**Table 5:** Measuring trial endpoints.

Outcome measures	Assessment
Percentage of Greater Manchester kidney physicians agreeing to enter patients into a study which includes a “higher range” group	Survey of the nephrology consultants in the Greater Manchester area

Percentage of eligible invited patients willing to be randomised into a study which includes a “higher range” group	Log of all eligible patients in the Greater Manchester area to be maintained

Percentage of patients achieving consistent control of serum phosphate in each group over a 10-month maintenance period	This information will be obtained from the trial database

Drop-out rate from the study due to adverse events, kidney transplantation, intercurrent illness, and death; these numbers will inform the power calculation for the larger national study	This information will be obtained from the trial database

Pill burden per patient to control serum phosphate	The total number of phosphate binding medications needed in every patient to achieve the desired range of serum phosphates will be calculated

Adherence with therapy	BAASIS once every 4 weeks

Willingness of subjects to participate in Communicare patient support programme	The number of patients willing to use the package will be documented

Mean symptom score assessed by Pittsburgh Dialysis Symptoms Index	This will be done at the beginning, midway, and the end of the study

Incidence of major vascular events, defined as nonfatal myocardial infarction or any cardiac death, any stroke, or any arterial revascularisation excluding dialysis access procedures	This information will be captured on the CRF and transferred to the trial database

## References

[1] Wagner M., Ansell D., Kent D. M. (2011). Predicting mortality in incident dialysis patients: an analysis of the United Kingdom renal registry. *American Journal of Kidney Diseases*.

[2] Wikström B., Jacobson S. H., Bragg-Gresham J., Eichleay M., Pisoni R., Port F. (2010). Dialysis outcomes and practice patterns study estimate of patient life-years attributable to modifiable haemodialysis practices in Sweden. *Scandinavian Journal of Urology and Nephrology*.

[3] Karamanidou C., Clatworthy J., Weinman J., Horne R. (2008). A systematic review of the prevalence and determinants of nonadherence to phosphate binding medication in patients with end-stage renal disease. *BMC Nephrology*.

[4] Chiu Y.-W., Teitelbaum I., Misra M., de Leon E. M., Adzize T., Mehrotra R. (2009). Pill burden, adherence, hyperphosphatemia, and quality of life in maintenance dialysis patients. *Clinical Journal of the American Society of Nephrology*.

[5] Block G. A., Hulbert-Shearon T. E., Levin N. W., Port F. K. (1998). Association of serum phosphorus and calcium x phosphate product with mortality risk in chronic hemodialysis patients: a national study. *The American Journal of Kidney Diseases*.

[6] Block G. A., Klassen P. S., Lazarus J. M., Ofsthun N., Lowrie E. G., Chertow G. M. (2004). Mineral metabolism, mortality, and morbidity in maintenance hemodialysis. *Journal of the American Society of Nephrology*.

[7] Kidney Disease: Improving Global Outcomes (KDIGO) CKD-MBD Work Group (2009). KDIGO clinical practice guideline for the diagnosis, evaluation, prevention, and treatment of Chronic Kidney Disease-Mineral and Bone Disorder (CKD-MBD). *Kidney International. Supplement*.

[8] Covic A., Kothawala P., Bernal M., Robbins S., Chalian A., Goldsmith D. (2009). Systematic review of the evidence underlying the association between mineral metabolism disturbances and risk of all-cause mortality, cardiovascular mortality and cardiovascular events in chronic kidney disease. *Nephrology Dialysis Transplantation*.

[9] Tentori F., Blayney M. J., Albert J. M. (2008). Mortality risk for dialysis patients with different levels of serum calcium, phosphorus, and PTH: the Dialysis Outcomes and Practice Patterns Study (DOPPS). *American Journal of Kidney Diseases*.

[10] Jamal S. A., Vandermeer B., Raggi P. (2013). Effect of calcium-based versus non-calcium-based phosphate binders on mortality in patients with chronic kidney disease: an updated systematic review and meta-analysis. *The Lancet*.

[11] Chertow G. M., Raggi P., McCarthy J. T. (2003). The effects of sevelamer and calcium acetate on proxies of atherosclerotic and arteriosclerotic vascular disease in hemodialysis patients. *The American Journal of Nephrology*.

[12] Chertow G. M., Burke S. K., Raggi P. (2002). Sevelamer attenuates the progression of coronary and aortic calcification in hemodialysis patients. *Kidney International*.

[13] Russo D., Miranda I., Ruocco C. (2007). The progression of coronary artery calcification in predialysis patients on calcium carbonate or sevelamer. *Kidney International*.

[14] Braun J., Asmus H.-G., Holzer H. (2004). Long-term comparison of a calcium-free phosphate binder and calcium carbonate—phosphorus metabolism and cardiovascular calcification. *Clinical Nephrology*.

[15] Barreto D. V., Barreto F. D. C., de Carvalho A. B. (2008). Phosphate binder impact on bone remodeling and coronary calcification—results from the BRiC study. *Nephron Clinical Practice*.

[16] Qunibi W., Moustafa M., Muenz L. R. (2008). A 1-year randomized trial of calcium acetate versus sevelamer on progression of coronary artery calcification in hemodialysis patients with comparable lipid control: the Calcium Acetate Renagel Evaluation-2 (CARE-2) Study. *American Journal of Kidney Diseases*.

[17] Suki W. N. (2008). Effects of sevelamer and calcium-based phosphate binders on mortality in hemodialysis patients: results of a randomized clinical trial. *Journal of Renal Nutrition*.

[18] Takei T., Otsubo S., Uchida K. (2008). Effects of sevelamer on the progression of vascular calcification in patients on chronic haemodialysis. *Nephron Clinical Practice*.

[19] Parham R., Riley S., Hutchinson A., Horne R. (2009). Patients' satisfaction with information about phosphate-binding medication. *Journal of Renal Care*.

[20] Webb L., Casula A., Ravanan R., Tomson C. R. V. (2010). UK Renal Registry 12th Annual Report (December 2009): chapter 5: demographic and biochemistry profile of kidney transplant recipients in the UK in 2008: national and centre-specific analyses.. *Nephron. Clinical practice*.

[21] Tomson C. R. V. (2010). UK Renal Registry 12th Annual Report (December 2009): chapter 1 summary of findings in the 2009 UK Renal Registry Report. *Nephron. Clinical Practice*.

[22] Toussaint N. D., Pedagogos E., Beavis J., Becker G. J., Polkinghorne K. R., Kerr P. G. (2011). Improving CKD-MBD management in haemodialysis patients: barrier analysis for implementing better practice. *Nephrology Dialysis Transplantation*.

[23] Covic A., Rastogi A. (2013). Hyperphosphatemia in patients with ESRD: assessing the current evidence linking outcomes with treatment adherence. *BMC Nephrology*.

[24] Shaw C., Pruthi R., Pitcher D., Fogarty D. (2013). UK renal registry 15th annual report: chapter 2 UK RRT prevalence in 2011: National and centre-specific analyses. *Nephron. Clinical Practice*.

[25] Hutchison A. J., Smith C. P., Brenchley P. E. C. (2011). Pharmacology, efficacy and safety of oral phosphate binders. *Nature Reviews Nephrology*.

[26] Isakova T., Gutierrez O. M., Chang Y. (2009). Phosphorus binders and survival on hemodialysis. *Journal of the American Society of Nephrology*.

[27] Weisbord S. D., Fried L. F., Arnold R. M. (2004). Development of a symptom assessment instrument for chronic hemodialysis patients: the Dialysis Symptom Index. *Journal of Pain and Symptom Management*.

[28] Baigent C., Landray M. J., Reith C. (2011). The effects of lowering LDL cholesterol with simvastatin plus ezetimibe in patients with chronic kidney disease (Study of Heart and Renal Protection): a randomised placebo-controlled trial. *The Lancet*.

[29] Rastogi A. (2013). Sevelamer revisited: pleiotropic effects on endothelial and cardiovascular risk factors in chronic kidney disease and end-stage renal disease. *Therapeutic Advances in Cardiovascular Disease*.

[30] Bays H. E., Goldberg R. B., Truitt K. E., Jones M. R. (2008). Colesevelam hydrochloride therapy in patients with type 2 diabetes mellitus treated with metformin: glucose and lipid effects. *Archives of Internal Medicine*.

[31] Schäfer-Keller P., Steiger J., Bock A., Denhaerynck K., De Geest S. (2008). Diagnostic accuracy of measurement methods to assess non-adherence to immunosuppressive drugs in kidney transplant recipients. *The American Journal of Transplantation*.

[32] Dobbels F., Berben L., De Geest S. (2010). The psychometric properties and practicability of self-report instruments to identify medication nonadherence in adult transplant patients: a systematic review. *Transplantation*.

[33] Doesch A. O., Mueller S., Konstandin M. (2010). Increased adherence after switch from twice daily calcineurin inhibitor based treatment to once daily modified released tacrolimus in heart transplantation: a pre-experimental study. *Transplantation Proceedings*.

